# Multiple-allergen oral immunotherapy improves quality of life in caregivers of food-allergic pediatric subjects

**DOI:** 10.1186/1710-1492-10-25

**Published:** 2014-05-12

**Authors:** Iris M Otani, Philippe Bégin, Clare Kearney, Tina LR Dominguez, Anjuli Mehrotra, Liane R Bacal, Shruti Wilson, Kari Nadeau

**Affiliations:** 1 Stanford Alliance for Food Allergy Research, Palo Alto, CA, USA; 2 Department of Pediatrics, Division of Immunology and Allergy, Stanford University, 269 Campus Drive, CCSR Building Suite 3215, Stanford, California 94305, USA

**Keywords:** Oral immunotherapy, Omalizumab, Anti-IgE, Food allergy, Oral desensitization, Quality of life, Health-related quality of life

## Abstract

**Background:**

Food allergy (FA) negatively affects quality of life in caregivers of food-allergic children, imposing a psychosocial and economic burden. Oral immunotherapy (OIT) is a promising investigational therapy for FA. However, OIT can be a source of anxiety as it carries risk for allergic reactions. The effect of OIT with multiple food allergens (mOIT) on FA-specific health-related quality of life (HRQL) has never been studied in participants with multiple, severe food allergies. This study is the first to investigate the effects of mOIT on FA-related HRQL in caregivers of pediatric subjects.

**Methods:**

Caregiver HRQL was assessed using a validated Food Allergy Quality of Life – Parental Burden (FAQL-PB) Questionnaire (J Allergy Clin Immunol 114(5):1159-1163, 2004). Parents of participants in two single-center Phase I clinical trials receiving mOIT (n = 29) or rush mOIT with anti-IgE (omalizumab) pre-treatment (n = 11) completed the FAQL-PB prior to study intervention and at 2 follow-up time-points (6 months and 18 months). Parents of subjects not receiving OIT (control group, n = 10) completed the FAQL-PB for the same time-points.

**Results:**

HRQL improved with clinical (change < -0.5) and statistical (p < 0.05) significance in the mOIT group (baseline mean 3.9, 95% CI 3.4-4.4; 6-month follow-up mean 2.5, 95% CI 2.0-3.0; 18-month follow-up mean 1.8, 95% CI 1.4-2.1) and rush mOIT group (baseline mean 3.9, 95% CI 3.1-4.7; 6-month follow-up mean 1.7, 95% CI 0.9-2.6; 18-month follow-up mean 1.3, 95% CI 0.3-2.4). HRQL scores did not significantly change in the control group (n = 10).

**Conclusion:**

Multi-allergen OIT with or without omalizumab leads to improvement in caregiver HRQL, suggesting that mOIT can help relieve the psychosocial and economic burden FA imposes on caregivers of food-allergic children.

## Introduction

Food allergy (FA) is an adverse immunologic response to dietary antigen with a reported prevalence of 8% in the United States. Approximately 30.4% of food-allergic participants are multi-sensitized, or allergic to more than one food allergen [1]. In Canada, the prevalence of pediatric food allergy has been reported to be 7.14% [2]. Currently, the only FDA-approved treatment for FA is food allergen avoidance and injectable epinephrine [3].

Food allergies have a significant negative impact on participants’ quality of life [4-6]. The burden of FA on caregivers (buying special foods, limiting social encounters, foregoing full-time employment) has been reported to play a predominant role in the total annual economic burden of FA, $24.8 billion [7]. Compared to those with single food allergies, those with multiple food allergies experience a greater decrease in quality of life [5,8], are more likely to suffer from dietary deficiencies [9] and are less likely to outgrow their food allergies [10].

Oral immunotherapy (OIT) is a promising investigational therapy for food allergy [11]. We recently conducted two Phase I clinical trials showing the safety and feasibility of 1) an OIT protocol for desensitization to multiple allergens simultaneously (mOIT) [12] and 2) a protocol combining anti-IgE therapy (omalizumab) and a rush mOIT schedule to allow a rapid desensitization (rush mOIT) [13].

Although it is intuitive to think that successful desensitization to multiple food allergens would improve quality of life, safety analyses have shown that allergic reactions are common with OIT dosing. Although the majority of reactions are reported as mild, events requiring injectable epinephrine do occur [12,13]. This raises the question as to whether reactions with dosing outweigh the benefit gained from therapy, which can only be addressed by an objective evaluation of quality of life influenced specifically by health and disease, or health-related quality of life (HRQL) [14-16]. While single-allergen OIT has been found to result in HRQL improvement in participants with peanut or cow milk allergy [17,18], this study is the first to investigate the effects of mOIT and of rush mOIT with omalizumab on FA-related HRQL.

## Methods

### Participants

Questionnaires were distributed to caregivers of 3 groups of participants: 2 experimental groups enrolled in 2 separate ongoing single-center Phase I clinical trials conducted under INDs with Stanford IRB approvals and 1 control group whose only intervention was food allergen avoidance. Written consent was obtained prior to study entry.

Eligibility criteria are described in detail in previous publications [12,13]. Briefly, in all the groups, participants older than 4 years were eligible for inclusion if they had proven sensitivity to their main food allergen documented by both a positive skin prick test specific IgE as well as positive allergic reaction in a double-blind placebo-controlled oral food challenge (DBPCFC) up to a cumulative dose of 182 mg as per Bock’s criteria [12,13]. To be included in the treatment, additional food allergens also needed to induce a reaction within a cumulative dose of 182 mg on DBPCFC.

### Study intervention

The intervention and medication used in the mOIT and rush mOIT groups have been described in detail previously [12,13]. Briefly, subjects meeting inclusion criteria were started on a daily dose of up to 5 multiple food allergens combined in an equivalent stoichiometric ratio (1:1:1:1:1) based on food protein content. In the mOIT group, participants underwent an initial dose escalation starting at 0.1 mg of total food protein up to a maximum of 6 mg if tolerated. The maximal tolerated dose determined daily home dose that was increased by 25% increments every other week at our research clinic based on dose tolerability. Participants took a median of 85 weeks to reach their maintenance dose of 4000 mg protein per food. In the rush mOIT group, participants underwent a 16-week omalizumab treatment, starting 8 weeks prior to rush mOIT. They underwent rush desensitization up to 1250 mg of total food protein on their first day, followed by similar biweekly increases in home doses. Final maintenance dose was reached at a median of 18 weeks.

### Questionnaire

Health-related quality of life (HRQL) was assessed using a validated Food Allergy Quality of Life – Parental Burden (FAQL-PB) Questionnaire [1]. The FAQL-PB is a FA-specific HRQL questionnaire that measures parental burden associated with having a child with FA. It was originally validated in the United States by Cohen et al. and consists of 17 questions that are answered on a 7-point scale.

All patients in the mOIT and the rush mOIT trials who started the protocol between January 1 and April 1 2012 were asked to complete the questionnaire prior to study intervention. The same parent who filled out the questionnaire at baseline completed the subsequent follow-up questionnaires at a 6-month follow-up time-point on OIT and an 18-month follow-up time-point. Only caregivers of participants aged less than 17-years were included to be consistent with the age group used in the validation phase of the FAQL-PB questionnaire by Cohen et al. [1].

During the same period, all consecutive subjects screened and meeting the same inclusion criteria as the mOIT and rush mOIT trials were asked to fill out the questionnaire. Those who were not included in the mOIT and rush mOIT trials despite meeting inclusion criteria due to lack of available space were asked to fill out follow-up questionnaires as an untreated control group.

### Statistics

Comparisons between HRQL scores were made with the Wilcoxon for paired variables and the Mann–Whitney test for unpaired variables, as appropriate. A 2-tailed p-value of less than 0.05 was considered statistically significant.

Clinical relevance was assessed using the MID, or minimal clinically important difference. The MID has previously been defined as the smallest change in HRQL that participants perceive as clinically important. It has been estimated to be approximately 0.5 for scores graded on a 7-point scale in several HRQL questionnaires, including questionnaires assessing parental quality of life [19-23].

The therapeutic value of oral immunotherapy was determined by calculating the number-needed-to-treat (NNT) as done previously by van der Velde et al. [24,25]. The proportion of participants who benefited from multi-allergen oral immunotherapy was calculated as the difference of the proportion of participants with clinically important improvement in HRQL in the mOIT group minus the proportion of participants with improvement in HRQL in the control group. The NNT is the reciprocal of the proportion of participants benefiting from OIT. All statistical analyses and graphing were carried out with GraphPad Prism software (GraphPad Software, San Diego, CA).

## Results

During the initial recruitment period for the HRQL study, 29 participants started the mOIT trial and 11 participants started the rush mOIT trial. Caregivers of all these participants completed the FAQL-PB questionnaire at all required time-points. During the same period, 24 participants were screened and met criteria for entry into an OIT trial at our center. All caregivers accepted to complete the questionnaire as controls. Fourteen were eventually included in a trial prior to 6-month follow-up and were thus excluded from analysis. Of the 10 remaining participants, 2 were lost to follow-up prior to the 18-month follow-up. Baseline characteristics were similar between all 3 groups (Table 1).

**Table 1:** Baseline demographics and clinical characteristics of pediatric subjects in the mOIT, rush mOIT, and control groups

	mOIT*	Rush mOIT*	**Control**
**Number of subjects**	29	11	10
**Median age in years (range)**	8 (4 – 13)	7 (4 – 16)	8 (4–14)
**Male**	18 (62%)	8 (72%)	5 (50%)
**Coexisting atopic disease**			
Atopic dermatitis	20 (69%)	5 (45%)	5 (50%)
Allergic rhinitis	15 (52%)	6 (55%)	6 (60%)
Asthma	21 (72%)	8 (73%)	7 (70%)
Food allergies meeting criteria for inclusion on DBPCFC**			
Peanut	20 (69%)	7 (64%)	7 (70%)
Walnut	11 (38%)	5 (45%)	4 (40%)
Cashew	9 (31%)	6 (55%)	3 (30%)
Pecan	8 (28%)	5 (45%)	3 (30%)
Milk	8 (28%)	3 (27%)	2 (20%)
Egg	4 (14%)	4 (36%)	3 (30%)
Sesame	4 (14%)	0 (0%)	2 (20%)
Almond	3 (10%)	3 (27%)	2 (20%)
Hazelnut	2 (7%)	1 (9%)	2 (20%)
**Average number of desensitized foods**			
	3	4	n/a
**Highest baseline food allergy test (median and range)**			
SPT in mm	13.5 (7–25.5)	10.5 (7–29.5)	10.5 (7–30.5)
Specific IgE in ku/L	82.5 (2.95- > 100)	36.4 (2- > 100)	61 (3.25- > 100)
Lowest amount triggering reaction in DBPCFC in mg protein	32.7 (0.1-182.7)	32.7 (0.1-182.7)	32.7 (0.1-182.7)

*OIT = oral immunotherapy. **DBPCFC = double blind placebo controlled food challenge.

**Figure 1:**
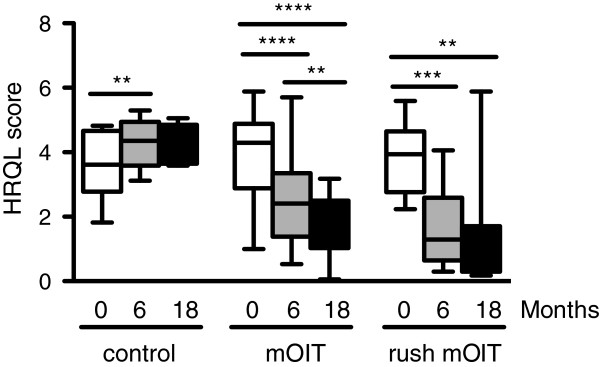
**HRQL scores improve in caregivers of participants on mOIT and rush mOIT.** Median HRQL score at baseline, 6-month follow-up, and 18-month follow-up are shown for the control group, mOIT group, and rush mOIT group. Whiskers represent minimum and maximum values. Median HRQL scores were 3.6, 4.4, 4.2 at baseline, 6 months, and 18 months, respectively, for the control group; 4.3, 2.4, 1.8 for the mOIT group; 3.9, 1.3, 0.8 for the rush mOIT group. ** p < 0.01, *** p = 0.001, **** p < 0.0001.

At baseline, all three groups had a high and comparable HRQL score (mean 3.6 to 3.9) out of a maximum of 6, indicating that all groups had a poor quality of life at baseline. Caregivers of participants in the mOIT group (n = 29) had a significant improvement in HRQL score at 6-month follow-up (mean 2.5, 95% CI 2.0-30) and 18-month follow-up (mean 1.8, 95% CI 1.4-2.1) compared to baseline (p < 0.0001). Caregivers of participants in the rush mOIT group (n = 11) also had a significant improvement in HRQL score at 6-month follow-up (mean 1.7, 95% CI 0.9-2.6) and 18-month follow-up (mean 1.3, 95% CI 0.3-2.4) compared to baseline (p = 0.001 and p = 0.005, respectively). HRQL worsened significantly (p < 0.01) in the control group (n = 10) from baseline (mean 3.6, 95% CI 2.9-4.3) to 6-month follow-up (mean 4.3, 95% CI 3.9-4.8). However, HRQL at 18-month follow-up (mean 4.3, 95% CI 3.7-4.7) was comparable to baseline HRQL in the control group (Figure 1). We did not find any association between the number of foods or adverse events and the change in HRQL score in either group although we may have lacked power for such sub-group analysis.

**Figure 2:**
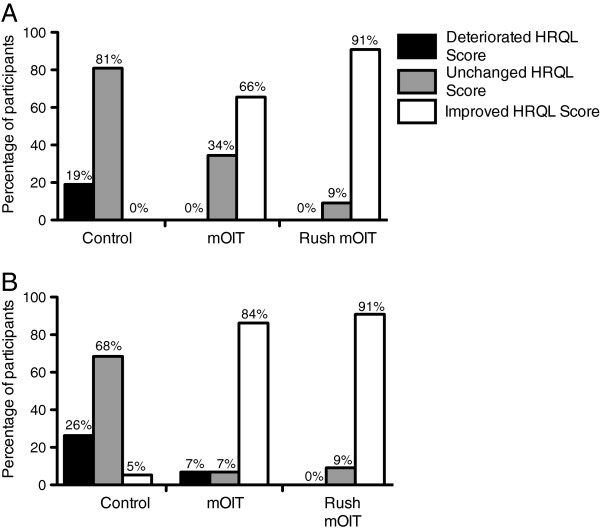
**Percentages of participants with deterioration, no change, or improvement in HRQL scores.** Percentages of participants whose HRQL scores deteriorated (change > 0.5), remained unchanged (change between -0.5 and 0.5), or improved (change < -0.5) in the control group, mOIT group, and rush mOIT group at **(A)** 6-month follow-up and **(B)** 18-month follow-up.

With both interventions, the improvement was clinically relevant (decrease greater than -0.5) at both time points (-1.9 and -2.5 at 6 and 18 months with mOIT, -2.6 and -3.1 at 6 and 18 months with rush mOIT). The percentages of participants with clinically relevant changes in FAQL-PB HRQL scores are shown for all 3 groups in Figure 2A (6-month follow-up) and Figure 2B (18-month follow-up).

To assess therapeutic value, we calculated the NNT to induce a clinically relevant improvement in QOL. At the 6-month follow-up time-point, NNT for mOIT was 1.5 and 1.1 for rush multi-allergen OIT. At the 18-month follow-up time-point, NNT was 1.2 for both multi-allergen OIT and rush multi-allergen OIT.

**Figure 3:**
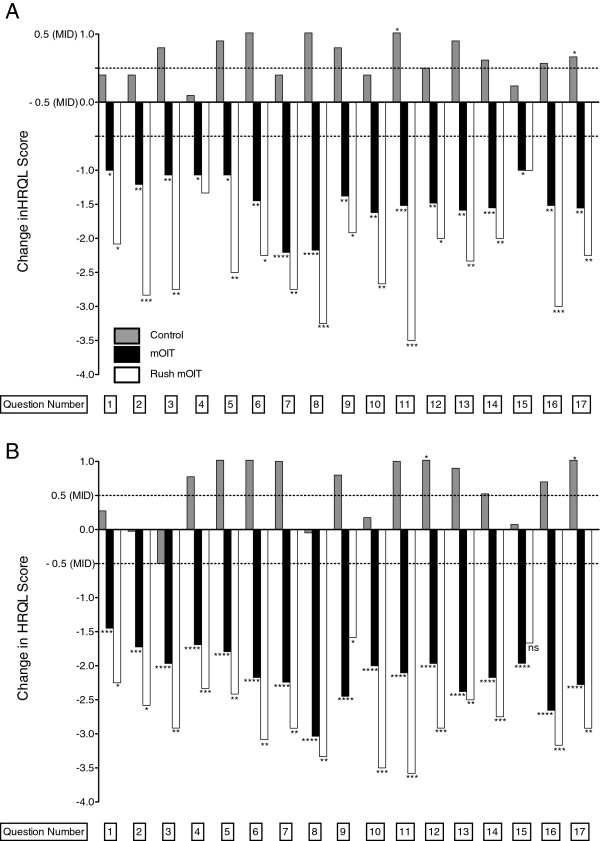
**Changes in individual FAQL-PB question scores.** Changes in individual FAQL-PB question scores are shown between and **(A)** 6-month follow-up and **(B)** 18-month follow-up. * p < 0.05, ** p < 0.01, *** p < 0.001, **** p < 0.0001. Bars without asterisks represent non-significant changes.

We looked at individual question scores to see if changes seen in overall HRQL scores were distributed across all questions or due to changes in specific question scores (Figure 3). In the mOIT group, caregivers’ answers showed a statistically significant (p < 0.05) and clinically relevant (change < -0.5) change for all 17 questions (Additional file 1: Table S1) at the 6-month follow-up that persisted at the 18-month follow-up. Similarly, in the rush mOIT group, caregivers’ answers showed a statistically significant and clinically relevant change for all but 2 questions at the 6-month and all but 1 question at the 18-month follow-up time-point.

## Discussion

Using a quality of life questionnaire specifically validated for food allergy, we have shown that two protocols for mOIT were associated with clinically and statistically significant increases in caregiver quality of life. This change persisted with 18 months of ongoing therapy and included all areas covered by the questionnaire. The effect was most pronounced after 18 months of rush mOIT combined with Omalizumab which showed a HRQL score decrease of 3.1 from median 3.9 to 0.8 (score ranked on a maximum of 6).

These findings are of major importance given the current question surrounding the role for OIT in the care for children with multiple food allergies. The efficacy of OIT is typically measured by the ability to tolerate food allergen after discontinuation of therapy (clinical tolerance or sustained unresponsiveness). Recent studies have shown limited rates of sustained unresponsiveness with OIT, raising concern about its relevance as an intervention for food allergy [26]. This study shows that desensitization itself, even without discontinuing therapy, provides significant and persistent improvement in caregiver quality of life. This suggests OIT can be of benefit to caregiver quality of life even in the absence of sustained unresponsiveness.

These results also suggest that the reaction profiles of mOIT and rush mOIT are acceptable and justified from a caregiver point-of-view and are much preferred to allergen avoidance. Home dosing reactions, which are expected and for which the patient is prepared may be less anxiety-producing than the constant fear of accidental reactions and uncertainty of day to day living with food allergy. In a review of 352 subjects desensitized to peanut, Wasserman and colleagues showed that severe reactions requiring epinephrine in the context of OIT were recognized and treated promptly and did not require additional intervention [27].

In addition to reducing reaction anxiety, the FAQL-PB questionnaire showed that mOIT had an impact on various aspects of day to day living associated with an economic burden for families with food allergy. These include arranging special diets ($1.7 billion spent annually in the United States) and avoidance of unintentional exposure to food allergens including childcare arrangements ($857 million), changing schools ($650 million), and attending special summer camps ($125 million) [8].

This study is the first to examine the effects of multi-allergen OIT protocols in caregivers of multi-sensitized participants. Two prior studies showed improvement in FA-specific HRQL with single-allergen OIT. These studies used a different questionnaire so their results cannot be directly compared to those presented here. To the best of our knowledge, the effect of single-allergen oral immunotherapy on HRQL has never been studied in participants with multiple, severe food allergies but one could assume that the effect would not be as drastic given it would only allow them to be less vigilant about one of their food allergens.

When comparing the two interventions, the magnitude of improvement was greater for the rush mOIT group, especially at the 6-month time point. Also, at 6-month follow-up, a larger percentage of participants had improved HRQL scores in the rush mOIT group (91%) when compared to the mOIT (66%) group, whereas at 18-month follow-up, the percentage of participants with improved HRQL scores was similar between the rush mOIT (91%) and mOIT (84%) group. The greater and more rapid HRQL improvement with rush mOIT probably reflects the fact that subjects reach maintenance much faster in this group (median 18 weeks) compared to mOIT without omalizumab (median 85 weeks) [12,13]. It is unlikely to be related to a protective effect of omalizumab as reaction rates were similar in both groups.

One limitation to consider is that all subjects were recruited from volunteers. Although this potentially introduced selection bias toward more severely affected families, this bias reflects the patient population that would seek out additional therapy such as oral immunotherapy. Also, these were Phase I studies. Although the control group was not placebo-controlled, it would not have been possible to test the full psychosocial effect of the intervention if subjects were blinded and did not know they were protected. Despite the control group being comparable and selected using the same criteria, it is possible that the intense follow-up with bi-weekly visits to see food allergy specialists during OIT escalation phase positively affected the treatment group caregiver quality of life. However, previous studies looking at allergist interventions such as DBPCFC (positive outcome) and self-regulation telephone intervention did not show significant impact on overall HRQL scores [22,28].

In conclusion, our findings suggest that mOIT, with or without omalizumab, can lead to significant improvements in caregiver HRQL that persist with ongoing treatment. These findings support OIT as a promising therapy for food allergy and suggest that OIT can help relieve the psychosocial burden food allergy imposes on caregivers of food-allergic children. Validated measures of quality of life should be included in future phase II clinical trials.

## Abbreviations

FA: Food allergy; OIT: Oral immunotherapy; mOIT: Oral immunotherapy with multiple food allergens simultaneously; HRQL: Health-related quality of life; FAQL-PB: Food allergy quality of life – parental burden.

## Competing interests

This project was approved by the IRB committee at Stanford University. The authors have no relevant conflict of interest to disclose.

## Authors’ contributions

KN conceived and designed the study. IO, CK, TD, AM, LB, and SW acquired data. IO analyzed the data. IO and PB interpreted the data. IO, PB, and KN drafted the manuscript. All authors revised the manuscript and approved the final version.

## Supplementary Material

Additional file 1: Table S1 Food Allergy Quality of Life-Parental Burden Questionnaire. Click here for file
